# Unveiling the Potential Role of Dhurrin in Sorghum During Infection by the Head Smut Pathogen *Sporisorium reilianum* f. sp. *reilianum*

**DOI:** 10.3390/plants14050740

**Published:** 2025-02-28

**Authors:** Coumba Fall, Seunghyun Lim, Ezekiel Ahn, Sunchung Park, Louis K. Prom, Clint W. Magill

**Affiliations:** 1 Department of Plant Pathology & Microbiology, Texas A&M University, College Station, TX 77843, USA; coumba@tamu.edu; 2 Sustainable Perennial Crops Laboratory, Beltsville Agricultural Research Center, Agricultural Research Service (ARS), United States Department of Agriculture (USDA), Beltsville, MD 20705, USA; 3 Southern Plains Agricultural Research Center, Agricultural Research Service (ARS), United States Department of Agriculture (USDA), College Station, TX 77845, USA

**Keywords:** cyanogenic glucoside, head smut, sorghum, single nucleotide polymorphism

## Abstract

The cyanogenic glucoside dhurrin is found in sorghum and has been reported for its role in defense against biotic and abiotic stresses, both involving hydrogen cyanide (HCN) release. The fungus *Sporisorium reilianum* f. sp. *reilianum* (SRS) causes sorghum head smut and the infection occurs at the seedling stage, later resulting in panicle loss. Here, the focus was to determine the role of dhurrin in sorghum’s reaction against SRS infection. We investigated the genomic basis of HCN potential (HCNp) variation and its relationship with seedlings’ response to SRS inoculation, along with other sorghum traits, and the expression of dhurrin biosynthetic genes in SRS-inoculated young sorghum. Genome-wide association studies (GWAS) using HCNp scores showed significant single nucleotide polymorphisms (SNPs) on chromosomes harboring the dhurrin biosynthetic and catabolic genes but not in proximity. Significant hits were also detected in or near genes encoding proteins involved in plant defense/resistance against biotic stresses. Correlation analyses showed a strong positive relationship between average HCNp scores and latent period in SRS-inoculated sorghum seedlings. RT-qPCR revealed that the dhurrin biosynthetic genes were upregulated in the leaves of the head smut resistant line BTx635 up to two days after SRS inoculation. Our results suggest the involvement of dhurrin in sorghum’s protection against SRS.

## 1. Introduction

Sorghum (*Sorghum bicolor* (L.) Moench) represents the fifth-most grown cereal crop in the world [1]. The crop’s ability to withstand water and nutrient-limited environments renders it particularly valuable in the present context of climate change [2]. As has been reported for 2500 other species [3], sorghum also contains a cyanogenic glucoside, namely, dhurrin. Cyanogenic glucosides are bioactive plant products [4] reported to play a role in plant defense against biotic and abiotic stresses [5]. Dhurrin is mainly produced by sorghum [6] and its biosynthesis pathway is the first described for a cyanogenic glucoside [7]. Dhurrin biosynthesis begins with the amino acid L-tyrosine as a substrate and involves the action of two membrane anchored cytochrome P450 enzymes (CYP79A1 and CYP71E1) in the first two steps, and a soluble uridine-diphosphate (UDP)-glucosyltransferase (UGT85B1) for the last step [4,8]. In the first step, CYP79A1 catalyzes the conversion of L-tyrosine to p-hydroxyphenylacetaldoxime [9,10]. Subsequently, this compound is converted to p-hydroxymandelonitrile in presence of CYP71E1 [11]. Finally, UGT85B1 catalyzes the last step of the biosynthesis, which corresponds to the conversion of p-hydroxymandelonitrile to a stable storage form: (S)-p-hydroxymandelonitrile-β-D glucopyranoside, i.e., dhurrin [12]. All the dhurrin biosynthetic genes are located on chromosome 1 [8]. For dhurrin, as for the other cyanogenic glucosides, the catabolic enzymes enabling their hydrolysis are stored in different compartments within the cells or in different tissues to prevent autotoxicity and are brought in contact upon tissue disruption (due to herbivores, pathogens, pests, freezing, and drought), causing hydrogen cyanide (HCN) release and thus corresponding to the bioactivation of the cyanogenic glucoside [4,13,14]. Dhurrin bioactivation has been reported to involve the dhurrinases DHR1, DHR2, DHR-like3, and DHR-like4, which are β-glucosidases, in addition to a hydrozynitrile lyase (HNL). The dhurrinase genes are located on chromosome 8, while the HNL is on chromosome 4 [6]. DHR1 and DHR2 have been reported to be isozymes [15]. Because dhurrin bioactivation involves the release of HCN, these two have long been considered as proxies of each other. Therefore, hydrogen cyanide potential (HCNp) has been used as an estimate of dhurrin in many studies, either quantitatively [16,17], semi-quantitatively [18,19], or qualitatively [20,21]. A recent study [22] that characterized the transcriptional regulation of the dhurrin pathway genes during sorghum development showed that the biosynthetic genes were highly expressed in all the young tissues, while the bio-activation genes, i.e., catabolic, were more expressed early in organ development with organ specific expression patterns. This suggests a dynamic expression of these genes as a function of the plant’s growth stage. In addition, it has previously been proposed that biosynthesis and catabolism play a role in the natural variation of leaf dhurrin content in different environments [23].

Among biotic stresses affecting sorghum, the facultative biotrophic fungus *Sporisorium reilianum* f. sp. *reilianum* (SRS) causes head smut. This disease can lead to complete panicle loss when successful infection occurs early in the season. Yield losses have been reported to reach 80% in highly infested fields [24]. At the beginning of the growing season, the germination of the sorghum seeds triggers the germination of the SRS teliospores which overwintered in the soil. Under conducive conditions, the pathogen enters the plant and colonizes the meristematic tissues [25]. The fungus then biotrophically lives in the plant and the symptoms will only appear at the panicle emergence stage, when the inflorescence is replaced by a sorus containing black masses of teliospores [26]. Another pathogen, *Colletotrichum sublineola*, the fungus causing sorghum anthracnose, is known to infect all the aerial parts of the crop [27]. As dhurrin has been reported for its role against biotic stresses, it would be of interest to investigate whether this compound is involved in sorghum’s response against the anthracnose and head smut pathogens.

In the present study, to verify that dhurrin and HCN are proxies of each other, we investigated the basis of HCN variation through genome-wide association study (GWAS) using HCNp scores of sorghum accessions/lines and a single nucleotide polymorphism (SNP) dataset based on their sequences. We then proceeded with analyses to shed light on the effect of HCNp on sorghum seed morphology traits and the crop’s response to the anthracnose and head smut pathogens. Lastly, the dhurrin biosynthetic genes’ expression after SRS inoculation on sorghum seedlings was studied.

## 2. Results

### 2.1. Distribution of HCNp Scores in Sorghum Accessions and Principal Component Analysis

**Figure 1:**
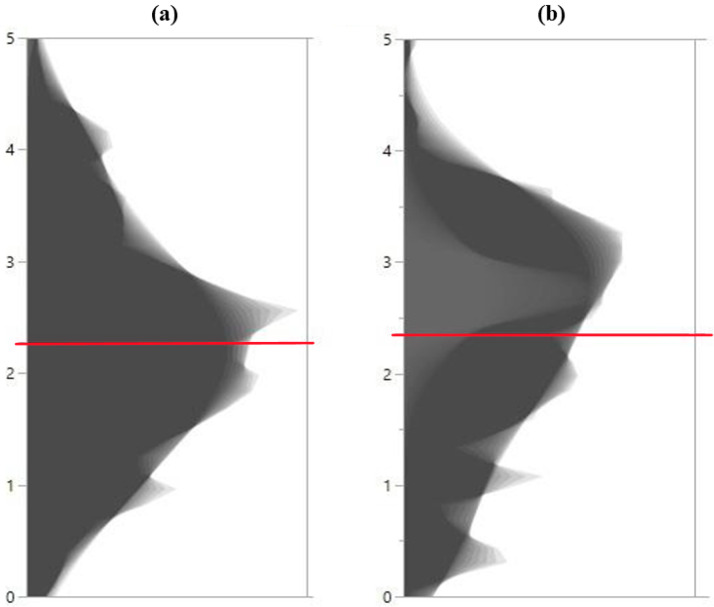
Distribution of hydrogen cyanide potential (HCNp) scores in sorghum accessions. Shadowgrams show the distribution of average HCNp scores in two sorghum populations. (**a**) A subset of the collection of Senegalese accessions from the National Plant Germplasm System (NPGS), C_1_ (average HCNp = 2.27) and (**b**) a subset of the collection composed of Nigerien and Senegalese accessions/lines, C_2_ (average HCNp = 2.36). The red lines represent the average HCNp value in each collection.

To investigate the basis of HCN variation in sorghum seedlings, HCNp release was first scored for accessions/lines from different collections, hereafter referred to as C_1_ (Senegalese accessions from the National Plant Germplasm System (NPGS), previously studied [28,29,30,31]) and C_2_ (Senegalese and Nigerien accessions [32]). For each accession/line, HCNp release was qualitatively assessed through visual scoring based on a previously reported semi-quantitative high throughput rating method [19], with some modifications. The HCNp was scored using a 0 to 5 scale as described in the ‘Materials and Methods’ section. In the original protocol [19], it was demonstrated that the visual scores provide results similar to those obtained from semi-quantitative measurements. The distributions of HCNp scores in the two sorghum collections (C_1_ and C_2_) are shown in Figure 1 as shadowgrams. The average score in a subset of the Senegalese collection, C_1_, (N = 126) was 2.27 (Figure 1a). In the newly collected Nigerien and Senegalese collection, C_2_, (N = 112), this score was 2.36 (Figure 1b), showing similar distributions across the two different groups. The SRS resistant (BTx635) and susceptible (BTx643) lines were, respectively, rated 2.67 and 4.25.

**Figure 2:**
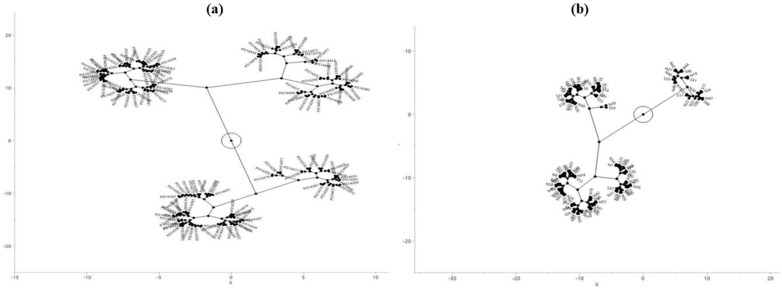
Constellation plots based on the average HCNp scores of the sorghum accessions/lines. (**a**) A subset of the Senegalese collection (C_1_); (**b**) 112 accessions/lines from Niger and Senegal (C_2_).

The Ward hierarchical clustering method was used to create constellation plots of HCNp scores to distinguish accessions in each of the two sorghum collections. In the subset of the Senegalese collection (C_1_), four major groups were formed (Figure 2a) while three in the recently collected Nigerien and Senegalese accessions/lines (C_2_) (Figure 2b), indicating slightly higher HCNp levels in the latter.

### 2.2. Correlation Between HCNp and Seed Morphology/Pathogen Inoculation Response Traits

Analyses were conducted to understand the relationship between the average HCNp scores (HCN.level) of accessions from C_1_ and their previously recorded seed morphology and pathogen inoculation response traits [28,29,30,31]. The latter traits consisted of: (i) the average time for spot appearance (HS.average.spot.appearance.rate) and the percent area covered by those spots (HS.average.spot.appearance.rate) in sorghum seedlings inoculated with a pathotype 5 of SRS [29]; and (ii) the average anthracnose severity (Seedling.anthracnose.average.score) and the highest scores (seedling.highest.score) recorded from excised sorghum leaves inoculated with a Texas isolate of *C. sublineola* at the one-leaf stage [30], in addition to the anthracnose severity scores of C_1_ accessions inoculated previously with a mixture of *C. sublineola* isolates at the eight-leaf stage in greenhouse settings [28]. The seed morphology traits were recorded from seeds of C_1_ accessions, and included: the area size (area.size (mm^2^)), the perimeter length (perimeter.length (mm)), the length (mm), the width (mm), the length to width ratio (length.width.ratio), the circularity (0–1 scale, where 0 = not circular and 1 = complete circle), the distance between the intersection of length and width of the seed (IS) and the center of gravity (CG), and the brightness (0–255 scale, where 0 = complete black, and 1 = pure white) of the seeds [31].

**Figure 3:**
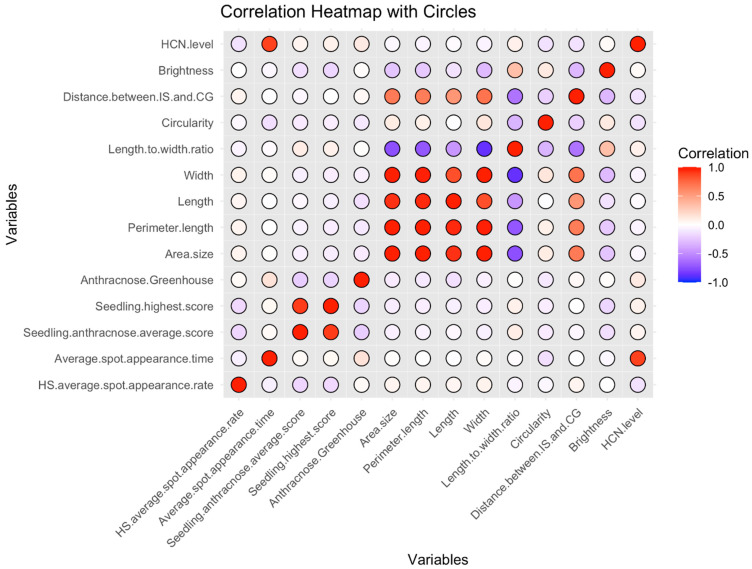
Heatmap of correlations between HCNp levels (scores) and other traits in Senegalese sorghum accessions from C_1_ The heatmap shows Pearson correlation coefficients between average HCNp scores and other traits, including seed morphology, resistance to anthracnose, average spot appearance rate, and time for spot appearance after inoculation with *Sporisorium reilianum* f. sp. *reilianum* (SRS). Red circles represent positive correlations, while blue circles represent negative correlations. The intensity of the color corresponds to the strength of the correlation. HCNp level showed a strong positive correlation (0.88) with time for spot appearance (*p*-value = 0.0001). No significant correlations were detected between HCNp levels and other traits.

Pearson’s correlation analysis between average HCNp scores of accessions from C_1_ and the listed traits, as shown in Figure 3, revealed a strong positive correlation between HCNp scores and the average time for spot appearance after sorghum seedlings’ inoculation with SRS (*p*-value = 0.0001, Pearson’s coefficient = 0.88). This demonstrates that sorghum HCNp is closely or even directly related to the development of the spots at the seedling stage. However, no statistically significant correlations were found between average HCNp scores and other previously studied traits, such as seed morphology and resistance to anthracnose.

**Figure 4:**
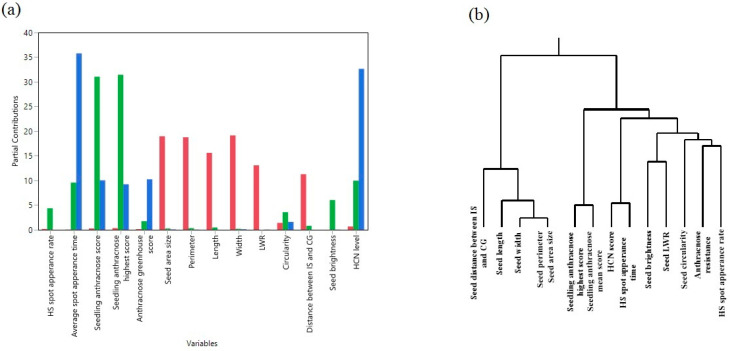
Relationship between HCNp levels (scores) and other traits in C_1_ accessions. (**a**) Visualization of how phenotypic variables contribute to the top three principal components (PCs). Red bars represent PC1, green bars PC2, and blue bars PC3. (**b**) Hierarchical clustering based on the Ward method among the traits.

The close relationship between HCNp levels and time for spot appearance after SRS inoculation, two traits that, a priori, do not seem to be associated with each other, are further confirmed by principal component (Figure 4a) and hierarchical cluster analyses. The hierarchical cluster analysisbased on the Ward method among the traits, generated a dendrogram (Figure 4b). Both plots (partial principal component analysis (PCA) plot (Figure 4a), dendrogram (Figure 4b)) agree that HCNp levels and spot appearance time are nearly identical.

**Figure 5:**
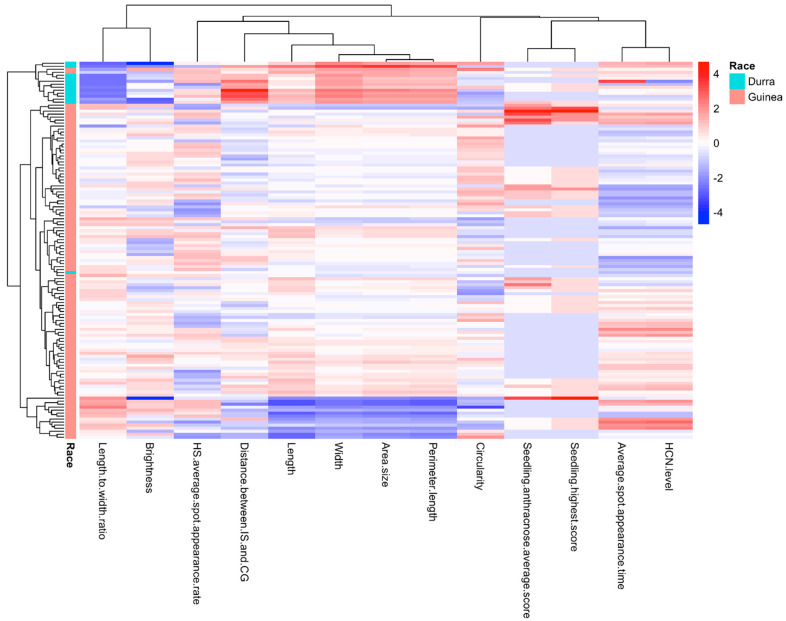
Heatmap of the HCNp levels’ (scores) and other traits’ distribution throughout the C_1_ collection. The heatmap displays various phenotypic distributions for each trait. The subset of Senegalese accessions was mostly composed of the Guinea race. The dendrogram above the heatmap illustrates the relationships between the traits.

The heatmap in Figure 5 illustrates the distribution of HCNp scores and other traits within the Senegalese sorghum accessions from C_1_. Hierarchical cluster analysis was performed using the average linkage method and the correlation coefficient as the similarity metric. The result again confirms the nearly identical pattern in the heatmap between HCNp scores and spot appearance time upon SRS inoculation, which only differed significantly in one sorghum accession (PI 514279 Race: Durra). The subset of Senegalese accessions was mainly composed of the Guinea race. The dendrogram above the heatmap illustrates the relationships between the different traits, similar to the dendrogram based on hierarchical clustering in Figure 4b.

### 2.3. Distribution and Characteristics of SNPs in the C_2_ Accessions/Lines from Niger and Senegal

**Figure 6:**
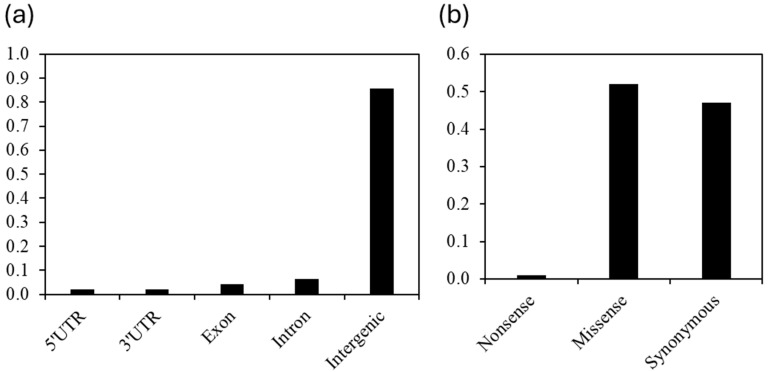
Distribution and functional impact of single nucleotide polymorphisms (SNPs) in the Sorghum genome. (**a**) Distribution of SNPs across the genome based on the gene models in the Sorghum reference genome. (**b**) Functional classification of the exonic SNPs. Synonymous SNPs do not change the amino acid sequence of the protein, while non-synonymous SNPs (including nonsense and missense mutations) can potentially alter protein function.

A SNP dataset was generated from the sequences of the C_2_ accessions/lines as described in the ‘Materials and Methods’ section. These SNP loci were annotated based on predicted gene models from the *S. bicolor* reference genome [33] to assess their impact on protein sequences using SNPeff [34]. Nearly 86% of the identified SNP loci resided within intergenic regions, while the remaining 16% were located within genes. Among genic SNPs, 26% (n = 30,104) were found in exons, and 44% (n = 46,324) were located within introns (Figure 6). Exonic SNPs were further classified based on their predicted effect on amino acid sequences. Approximately half (n = 15,978) of the exonic SNPs were predicted to cause amino acid changes (i.e., nonsynonymous substitutions), including 302 SNPs that introduced premature stop codons (i.e., nonsense) (Figure 6). The remaining half (n = 14,156) were synonymous substitutions. Considering all possible substitutions across codons are equally probable, a nonsynonymous substitution rate of 76% would be expected by chance [35]. Therefore, the observed lower rate of nonsynonymous substitutions suggested a strong selective pressure against such changes in the genes.

### 2.4. GWAS

**Figure 7:**
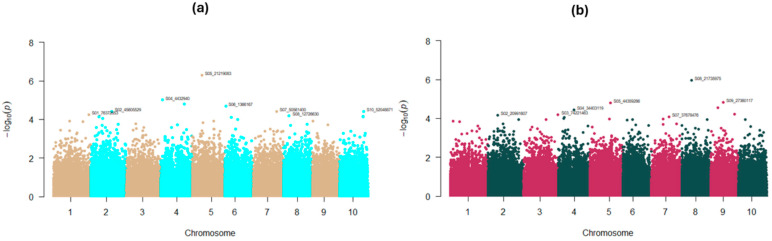
Manhattan plots showing significant SNPs detected through GWAS following the GLM procedure with: (**a**) average HCNp scores of 112 sorghum accessions/lines from C_2_, (**b**) average HCNp scores above 2.55, used as phenotypic data. SNPs with *p*-values smaller than 0.0001 are annotated. The corresponding QQ-plots are available in supplemental Figures S1 and S2.

To elucidate the genomic basis of the observed HCNp release variation, GWAS analyses were performed. Using the rank normalized average HCNp scores of 112 accessions from C_2_, GWAS was conducted following the GLM model in TASSEL (5.2.94) with the principal components of the SNP dataset used as covariates. SNPs with *p*-values smaller than 0.0001 were annotated (Figure 7a). Significant SNPs were detected at all chromosomes, except S03 and S09. The highest hit was detected on chromosome 5. However, S05_21,219,083 traced back to an unannotated genomic region. A significant SNP, S01_76,372,653, was found on chromosome 1 but not in the vicinity of any of the dhurrin biosynthetic genes with distances around 75 Mbp (*CYP79A1* ≈ 75.2 Mbp; *CYP71E1* ≈ 75.3 Mbp; *UGT85B1* ≈ 75.2 Mbp; all upstream). This long distance suggests that this SNP is unlikely to be in linkage disequilibrium (LD) with any allele in these loci, considering that Hamblin et al. [36] reported LD for sorghum to be largely decayed by 15 kb in their study. Nonetheless, S01_76,372,653 mapped back to *Sobic.001G445600.1* which encodes a protein similar to an exonuclease. Exonucleases are involved in DNA (deoxyribonucleic acid) replication, cell death, maintenance of genome stability, and immune responses [37]. This SNP was also near 8 kb upstream of *Sobic.001G445700.1*, which codes for a protein similar to β-glucosidase. β-glucosidases have been reported to be involved in cyanogenesis, during which a specific β-glucosidic enzyme hydrolyses the β-glucosidic linkage of the cyanogenic monoglucoside, thus producing an unstable α-hydroxynitrile that dissociates, causing the release of hydrogen cyanide [4]. The significant SNP found on chromosome 4, S04_4,432,940, mapped to *Sobic.004G053500*, which encodes for a protein similar to TIP120 (TBP (TATA-binding protein) -interacting protein 120). TIP120A has been reported to stimulate basal transcription ability [38]. In addition, S04_4,432,940 was found to be more than 60 Mbp upstream of the gene encoding the hydroxynitrile lyase (HNL) enzyme that has been reported to be involved in the catalysis of the dhurrin bioactivation process [6]. S06_1,366,167 traced back to *Sobic.006G008600*, which encodes a protein similar to H0201G08.11 that has a zinc-finger domain. Zinc-finger proteins form a superfamily of proteins involved in many plant processes, including the regulation of resistance mechanisms against biotic stresses. In fact, a zinc-finger domain has been reported in many disease resistance genes [39]. The significant SNPs detected on chromosomes 2, 7, 8, and 10, respectively, S02_45,605,529, S07_50,561.400, S08_12,726,630, and S10_52,048,871, were harbored in unannotated genomic regions. Although a significant SNP was detected on chromosome 8, within which are located the dhurrin catabolic genes (*DHR1*, *DHR2*, *DHR-like3*, *DHR-like4*), this SNP was far from those genes (635 kb–1 Mbp downstream).

A second GWAS was run using HCNp scores above 2.55 as phenotypic data (Figure 7b). The analysis was performed as described previously; more details are given in the ‘Materials and Methods’ section. The top SNP hit was detected on chromosome 8 (S08_21,735,975). However, the genomic region harboring this SNP was unannotated. S08_21,735,975 was far away (≈7–8 Mbp) from the dhurrinase coding genes. The significant SNPs identified on chromosomes 2, 3, 4, 5, and 7 (S02_20,991,607; S03_74,221,463; S04_34,403,119; S05_44,359,266; and S07_37,678,476) also mapped back to unannotated regions. Interestingly, S04_34,403,119 was closer to the HNL coding gene than S04_4,432,940, but still far (≈32.4 Mbp). S03_74,221,463 was near 1.4 kb upstream *of Sobic.003G360900.1*, which encodes for a protein similar to a cytochrome P450 present in rice. This gene corresponds to *OsCYP81Q32*, which has been reported to enhance resistance to mesosulfuron-methyl (a herbicide) when overexpressed in rice [40], thus likely playing a role in the detoxification of the compound. The first two enzymes involved in dhurrin biosynthesis are cytochrome P450s. In addition, cytochrome P450s have been reported to play a role in plants’ protection against diseases [41]. Near 4.2 kb downstream of S03_74,221,463, *Sobic.003G36800.1* was found. This gene encodes a protein similar to peroxidase-like protein. Peroxidases have been reported to be involved in plants’ defense against pathogens [42]. S09_27,360,113 traced back to *Sobic.009G094700.1*, which encodes for a protein similar to haloacid dehalogenase-like hydrolase (HAD) superfamily hydrolase. The HAD superfamily has been reported to play a role in abiotic stress response [43].

### 2.5. Expression of the Dhurrin Biosynthetic Genes upon Inoculation with SRS

The previous results showed HCNp as a potentially determining factor in sorghum’s defense against SRS. To find out how this relates to dhurrin biosynthesis, the expression of the genes involved in the pathway was investigated upon inoculation of sorghum seedlings with an SRS isolate.

**Figure 8:**
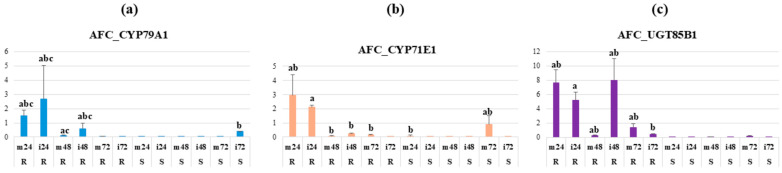
Average fold change (AFC) of the dhurrin biosynthetic genes upon mock or SRS inoculation of young BTx635 (SRS resistant (R)) and BTx643 (SRS susceptible (S)) plants: (**a**) *CYP79A1*; (**b**) *CYP71E1*; (**c**) *UGT85B1*. Letters above the error bars are based on the Games-Howell test (α = 0.05), bars sharing the same letter are not significantly different. m24R: BTx635 mock inoculated 24 h post-inoculation (hpi); m24S: BTx643 mock inoculated 24 hpi; m48R: BTx635 mock inoculated 48 hpi; m48S: BTx643 mock inoculated 48 hpi; m72R: BTx635 mock inoculated 72 hpi; m72S: BTx643 mock inoculated 72 hpi; i24R: BTx635 SRS inoculated 24 hpi; i24S: BTx643 SRS inoculated 24 hpi; i48R: BTx635 SRS inoculated 48 hpi; i48S: BTx643 SRS inoculated; i72R: BTx635 SRS inoculated 72 hpi; i72S: BTx643 SRS inoculated 72 hpi.

The head smut resistant and susceptible lines, respectively, BTx635 and BTx643, were syringe-inoculated with SRS. Mock inoculations were also performed to serve as controls. RT-qPCR analysis was then run on samples taken before inoculation, as well as one, two, and three days after. Using the cycle threshold values (CT), the relative fold change for each of the dhurrin biosynthetic genes, each tested line, at each time point and for each inoculum type (mock/SRS), was calculated. *Actin* was used as the reference gene. Figure 8 shows the calculated average relative fold change (AFC) of each of the dhurrin biosynthetic genes 24, 48, and 72 h post-inoculation (hpi). The results are shown for both mock (m) and SRS (i) inoculated plants. All genes were upregulated 24 hpi in both mock and SRS-inoculated head smut-resistant plants (BTx635), suggesting a possible induction due to the syringe inoculations. However, the AFC of *CYP79A1* almost doubled in the SRS-inoculated plants (≈2.7-fold) as compared to the mock (≈1.5-fold), and the enzyme encoded has been reported to have high substrate specificity for tyrosine [44], reinforcing the idea that dhurrin biosynthesis was triggered upon inoculation. In addition, *UGT85B1* was expressed at a much higher rate (≈8-fold), up to 48 hpi in the SRS-inoculated BTx635 plants, whereas almost no expression was found in the mock-inoculated plants. The genes showed almost no expression in the SRS-susceptible plants (BTx643). These suggest that dhurrin biosynthesis was not only triggered by syringe-induced wounds, but also by SRS inoculation. Therefore, dhurrin may actually serve as a protection for sorghum seedlings against successful SRS infection.

## 3. Discussion

The goal of the present study was to enlighten the genomic basis of HCNp variation in sorghum seedlings and the relationship between this compound and other sorghum traits, in addition to the role of dhurrin in sorghum’s protection against SRS infection. For this purpose, HCNp was scored for accessions/lines from two different collections as mentioned above and described in the ‘Material and Methods’ section. The average HCNp scores in both studied collections were between 2 and 3. However, accessions/lines with extreme values were present in both. The SRS resistant line BTx635 showed a high HCNp average score as compared to the susceptible line BTx643. Based on the Ward hierarchical clustering results, HCNp seems to allow the grouping of the accessions/lines, suggesting it as a potential way to categorize them. Using the average HCNp scores of accessions from C_1_ and their previously reported seed morphology/pathogen inoculation response traits, a correlation study was performed. The analysis showed a strong positive correlation between the latent period upon seedling inoculation with SRS and the average HCNp scores. To our knowledge, this is reported for the first time and needs further investigation. On the other hand, no correlation was found between those HCNp scores and seed morphology traits, nor anthracnose severity. For the seed morphology traits, the absence of correlation might be due to low dhurrin content in sorghum seeds. In fact, it has been reported that during maturation, dhurrin is replaced by proanthocyanidins, which act as defense compounds [45]. Regarding the anthracnose severity, it has been demonstrated that sorghum’s defense mechanism against *C. sublineola* infection involves luteolinidin and apigeninidin, which are phytoalexins from the flavonoid biosynthesis pathway [46].

To gather information about the SNPs generated from the C_2_ accessions’ sequences, the dataset has been explored. The prediction of the SNPs’ positions in the sorghum reference genome (GCF_000003195.3) and their functional effects showed that most of them (≈80%) were within intergenic regions. Half of the exonic SNPs were predicted to be nonsynonymous substitutions, but their rate was lower than the expected value, suggesting that the genes harboring them are likely to code for proteins playing a role in the organism’s fitness. However, the number of nonsynonymous substitutions was higher than the synonymous (≈1800), with most of them predicted to be missense, thus likely deleterious. It has been reported that some deleterious mutations are associated with heterosis, genetic variation, and key loss-of-function domestication traits underpinning crop production [47]. Investigating further the functional impact of the predicted missense SNPs would be interesting, as it has been suggested that the knowledge of deleterious mutations could help mitigate their effects through genome editing or conventional breeding [48].

A curated SNP dataset from the C_2_ accessions was used to perform two GWAS using all the HCNp scores of the subset studied (112 accessions) or those that were above 2.55. Both analyses revealed significant SNPs on chromosomes 1 (GWAS using all the average HCNp scores), 4 (both), and 8 (both), which harbor the dhurrin biosynthetic (chromosome 1) and catabolic (chromosomes 4 and 8) genes. However, these SNPs’ locations were far from those genes. Many of the significant SNPs detected traced back to genomic regions harboring genes coding for proteins that have been reported for their role in plants’ defense against biotic and abiotic stresses. Significant SNPs were also mapped back to genes encoding protein motifs associated with disease resistance (zinc finger). A significant SNP identified on chromosome 4 (GWAS using all the C_2_ HCNp scores) was localized within a gene coding for a protein similar to TIP 120. TIP 120 is a bipartite transcription factor that has been isolated from rat nuclear extracts, with both of its N- and C- terminal regions reported to have TBP (TATA-binding protein)-binding activity, in addition to being able to stimulate basal transcription ability [38]. This finding needs further investigation, considering that one of the dhurrin catabolic genes, the HNL coding gene, is located on the same chromosome. In fact, little is known about the molecular regulation of the dhurrin biosynthetic genes [49] and this seems to be the same for the catabolic genes. A negative candidate regulator of *SbCYP79A1*’s expression, *SbGATA22*, has recently been identified. *SbGATA22* is an LLM domain B-GATA transcription factor which binds to the putative GATA transcription factor, binding motifs within the *SbCYP79A1* promoter region [49]. The authors highlighted that this transcription factor needed further characterization to confirm whether it directly controls *SbCYP79A1*’s expression or requires the action of cofactors.

Based on the positive correlation found between the average HCNp scores of the accessions from C_1_ and average time for symptom appearance upon inoculation of their seedlings, a gene expression study using RT-qPCR was performed to investigate the dhurrin biosynthetic genes’ regulation once seedlings (growth stages 1–2) of SRS-resistant and susceptible lines are inoculated with this pathogen. In the SRS-resistant background, all the dhurrin biosynthetic genes were upregulated upon inoculation of the BTx635 plants with SRS 24 hpi. However, the genes encoding for the first two enzymes were downregulated 48 hpi, whereas UGT85B1 coding gene was more expressed at that time before going down the following day. This enzyme has been shown to be required for dhurrin synthesis in sorghum without a co-expressed UDP-glucosyltransferase to substitute it [50]. Regarding SRS, assuming that HCN is the compound that disables its infection on sorghum, this fungus is probably not able to detoxify HCN as it has been reported for other fungi [51] such as *Microdochium sorghi* (formerly *Gloeocercospora sorghi*, fungal pathogen causing zonate leaf spot on sorghum), which is less sensitive to HCN through the action of its endogenous cyanide hydratase enzyme [52]. In addition, it has previously been shown that the inoculation of BTx635 seedlings with SRS did not cause a change in the *PAL* (phenylalanine ammonia-lyase) nor the *CHS* (chalcone synthase) genes [51], although these genes have previously been reported to be upregulated upon sorghum inoculation with other pathogens. Indeed, PAL transcripts have been demonstrated to accumulate in sorghum mesocotyls inoculated with the nonhost *Bipolaris maydis* [53]. In silico analysis also previously revealed a *CHS*-like gene, *CHS8*, that showed significantly higher expressed sequence tags (EST) in *Colletotrichum sublineola*-induced library [54]. As opposed to their expression in SRS-resistant plants, the dhurrin biosynthetic genes lacked expression in the susceptible background (BTx643). These suggest that dhurrin might play a role in sorghum seedlings’ defense against SRS invasion through its bioactivation and the release of HCN. This assumption will need experimental proof; thus, investigating how the dhurrin catabolic genes are regulated upon SRS inoculation would help confirm the present results and determine the role of HCN in sorghum’s defense against this pathogen.

## 4. Materials and Methods

### 4.1. Plant Material

Two sorghum collections, C_1_ and C_2_, were used in this study. C_1_ comprised 163 Senegalese sorghum accessions from the National Plant Germplasm System (NPGS) and C_2_ comprised 120 accessions/lines from Niger and Senegal (60 from each country). Accessions/lines from both collections were scored for their HCNp as described below (4.3), and the collected data were used in correlation analyses (C_1_) and GWAS (C_2_) Additional information regarding these collections is available in supplementary data (Table S1). The sorghum lines BTx635 and BTx643 were used for the dhurrin biosynthetic genes’ expression study. Maize PI587124 from the NPGS was used as the negative control for HCNp visual scoring, as there is not yet evidence of this crop being cyanogenic, though a study has shown that the crop produces aldoximes following herbivory simulation [55]. In fact, aldoximes are intermediate compounds in cyanogenic glucoside biosynthesis. These authors also proceeded with a comparative analysis of the sorghum and maize genomes, but did not find an ortholog of *CYP79A1* in the latter. Thus, they hypothesized that the gene might have been lost, which could explain the absence of dhurrin, and therefore HCN formation, in maize.

### 4.2. Fungal Material

The SRS isolates were recovered from infected panicles. The isolate used in the correlation studies was a pathotype 5 [29], whereas pathotype determination has not been conducted yet for the one (HS132) used in the gene expression study. The *C. sublineola* isolates used for inoculations previously [28,30] were all from Burleson County in Texas.

### 4.3. HCNp Rating

**Figure 9:**
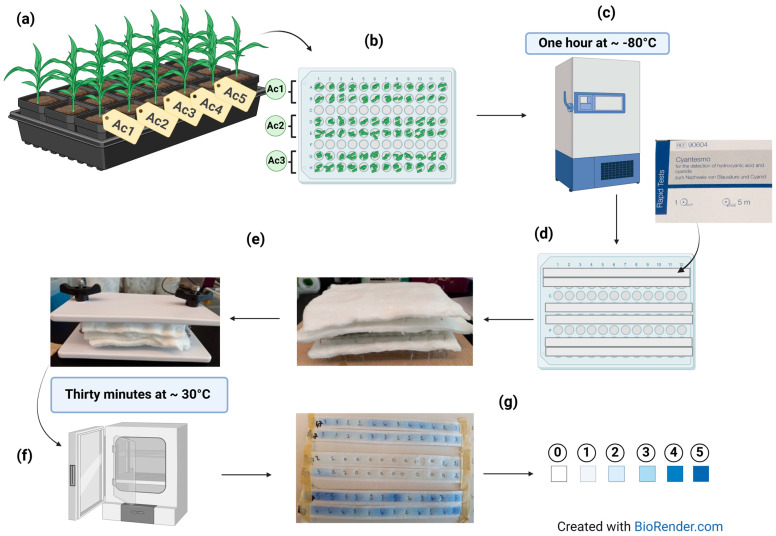
HCNp rating protocol: (**a**) sorghum plants used (Ac followed by a number is an abbreviation for the accession/line name); (**b**) leaf fragments introduced into the 96-well plate with two rows per accession/line; (**c**) incubation at −80 °C for an hour; (**d**) wells containing leaf fragments covered with Cyantesmo paper strips; (**e**) plates covered with cotton layers and introduced in a tofu press to avoid leakage; (**f**) incubation at 30 °C for 30 min; and (**g**) Cyantesmo paper strips after incubation and rating scale with each square corresponding to a score, from the lowest (0) to the highest (5).

The protocol used was modified from Johnson [19] and is depicted in Figure 9. The plants were grown at room temperature in flat, plugged trays containing potting mix (Figure 9a). The scoring was performed once the plants were 8 to 15 days old. For each accession/line, the leaves were cut into pieces with scissors and introduced into a sterile 96-well plates. A total of 24 wells were filled with 2–4 leaf fragments using clean tweezers, depending on their size, and 12 wells were left blank between each two accessions to avoid interference (Figure 9b). The scissors and tweezers were wiped with 70% ethanol between samples to avoid contamination. The plates were kept on ice when ready before being transferred to a freezer set at −80 °C for an hour (Figure 9c). Following the freeze exposure, the 24 wells of each accession/line were covered with commercially available Cyantesmo paper strips (Macherey-Nagel, GmbH & Co. KG Germany, Ref 90604) that detect the presence of HCN by showing a blue color, where the intensity depends on the amount of gas released (Figure 9d). Each 96-well plate was then closed, and a layer of cotton was put on the lid before stacking it with another one or two plates. The stacked plates were then put in a tofu press which was tightly sealed to avoid HCN leakage (Figure 9e). The ensemble was then incubated at 33–35 °C for 30 min (Figure 9f). After incubation, the HCNp level was rated using a 0–5 scale, depending on the intensity of the blue color with 0 corresponding to the absence of blue (Figure 9g) and 5 to a dark blue spot (Figure 9g). The average score for the 24 wells of each accession/line was then calculated.

### 4.4. Gene Expression Study

#### 4.4.1. Head Smut Pathogen Isolation and Identification

Harvested teliospores of HS132 were washed separately with 70% ethanol before being suspended in sterile distillated water containing 100 µg/mL streptomycin and 100 µg/mL ampicillin. Following this, 50 µL of the suspension were plated on potato dextrose agar (PDA) containing 100 µg/mL streptomycin and 100 µg/mL ampicillin. The plate was incubated until single colonies appeared [56]. A sterile swab was used to scrape the colonies from the plate and inoculate them in 5 mL potato dextrose broth (PDB). The colonies were dispersed in the broth by vortex, then a serial dilution was performed (10^−1^ to 10^−3^). A total of 50 µL of each dilution, as well as the initial suspension, were plated on PDA containing 100 µg/mL streptomycin and 100 µg/mL ampicillin. The plates were then incubated until single colonies appeared. The resulting colonies were assumed to represent independent products of meiosis [57]. A single colony was scraped from the plate, using a sterile toothpick, and inoculated to a new PDA plate containing 100 µg/mL streptomycin and 100 µg/mL ampicillin. The colonies were allowed to grow for around five days before the genomic DNA was extracted using a modified CTAB based method [58,59]. The quantity and quality of the extracts were assessed using a Quickdrop spectrophotometer followed by a visualization on 1% agarose gel, respectively. The ITS 1 and 4 primers were used for Polymerase Chain Reaction (PCR) amplification of HS132. After sequencing, the isolate was identified using the Basic Local Alignment Search Tool (BLAST, version 2.15.0) of the National Center for Biotechnology Information (NCBI). The sequence identity was 98.71% with *Sporisorium reilianum* f. sp. *reilianum* strain SRS1_H2-8 (Genbank Accession number: LT795063.1), confirming the isolate’s identity. BLAST results are available in the supplemental data (Figure S3).

#### 4.4.2. Oligonucleotides

The sequences of *Actin* and the dhurrin biosynthetic genes were retrieved from Genbank using their accession numbers (*Actin*: XM_021463392.1 (updated version: NM_001426217.1), dhurrin biosynthetic genes: [11,12,60]). The primers and probes were synthesized at Biosearch Technologies (Petaluma, CA, USA). The sequences are available in the supplemental data (Table S2).

#### 4.4.3. Plants

Seeds of BTx635 (SRS resistant) and BTx643 (SRS susceptible) were surface disinfected (10% bleach (three minutes), 70% ethanol (one minute), sterile water (three washes)) and air-dried on paper towels before being sowed in plugged trays filled with sterilized potting mix. Plants were grown in a growth chamber (VWR diurnal growth chamber, model 2015) set at 28 °C with an 8h photoperiod.

#### 4.4.4. Inoculation and Sampling

**Figure 10:**
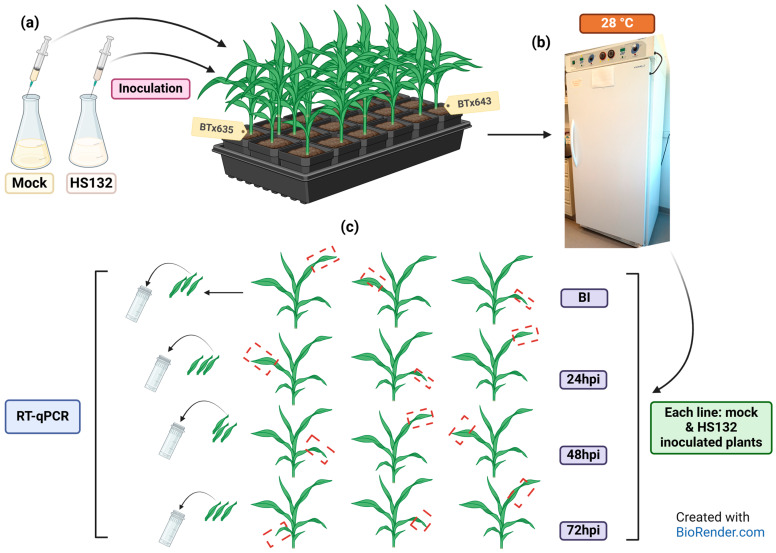
(**a**) SRS inoculation and sampling procedure: syringe inoculation of BTx635 and BTx643 plants with potato dextrose broth PDB (mock) and HS132 sporidial suspension (SRS); (**b**) incubation at 28 °C; (**c**) sampling method for RT-qPCR analysis: leaf fragments from three different plants were taken at different positions for each time, line, and inoculum type. BI = before inoculation; 24 hpi = 24 h post-inoculation; 48 hpi = 48 h post-inoculation; 72 hpi = 72 h post-inoculation.

A flask containing 100 mL of sterile PDB was inoculated with an agar plug (0.5 cm diameter) colonized by HS132 and shaken at room temperature until the suspension showed a milky color. The suspension was then filtered through a layer of sterile Miracloth (475855, Calbiochem-Novabiochem corporation). The concentration of the resulting filtrate was adjusted to 10^6^ sporidia/mL. Inoculation (Figure 10) was performed when plants were between growth stages 1 and 2. Beforehand, leaf samples were taken from three plants of each line, pooled, flash frozen with liquid nitrogen, and kept at −80 °C. (Figure 10c). Plants were then split into two batches for each line, excluding the sampled individuals. The first batch was inoculated with sterile PDB (mock) and the second with the HS132 sporidial suspension (Figure 10a). Inoculations were performed using sterile precision needles (PrecisionGlide needle, 25 G 5/8, Becton-Dickinson, Reorder No. 305122). For each plant, the needle was inserted into the stalk, below the first leaf. The inoculum was injected until droplets were coming out of the upper plant leaf sheaths. Inoculated plants were incubated in the dark at 28 °C overnight (inoculations performed between 6 and 7 p.m.) before the lights were turned back on (Figure 10b). Samples were taken from mock and head smut-inoculated plants of each line 24, 48, and 72 h post-inoculation (hpi). The same sampling method was used: three plants were sampled per line and inoculum type; each plant could only be sampled once during the experiment. Samples were flash frozen right after being collected and kept at −80 °C until RNA extraction (Figure 10c) to avoid degradation.

#### 4.4.5. RT-qPCR

RNA was extracted from samples using the Total RNA Mini Kit (Plant) from IBI Scientific (Cat. No. IB47341), following the manufacturer’s protocol. RT-qPCR was then conducted for each target gene using the One Step PrimeScript RT-PCR kit (Perfect Real Time) from Takara Bio Inc. (Cat. No. RR064A), as described by the manufacturer. For each gene, samples were repeated three times for each time point and each inoculum type (mock/HS132). Samples were run using a Smart Cycler II upgrade kit (Cepheid, model number: SC1000-1). Cycle threshold (CT) values were used to calculate the relative fold change for each gene, at each time point, following the 2^−∆∆Ct^ method [61]. *Actin* (*Actin-1*, LOC110436378) was used as the reference gene. The fold change (FC) was calculated as follows:

$${∆CT}_{t}={CT\left(target\right)}_{t}-{CT\left(reference\right)}_{t}$$

$${∆CT}_{t+k}={CT(target)}_{t+k}-{CT(reference)}_{t+k}$$

$${∆∆CT}_{t+k}={∆CT}_{t+k}-{∆CT}_{t}$$

$${FC}_{t+k}=2^{{-∆∆CT}_{t+k}}$$

where: ***t*** = before inoculation; ***t*** + ***k*** = either 24, 48, or 72 h post-inoculation; ***target*** = target gene (*CYP79A1*, *CYP71E1*, or *UGT85B1*); ***reference*** = reference gene (*Actin*).

### 4.5. SNP Dataset

Accessions of the C_2_ collection (120) were sequenced as described previously [32]. Leaf samples were collected between growth stages 1 and 2. The genomic DNA was then extracted, and sequencing was performed using the Illumina NovaSeq 6000 platform. The raw data sequences are also available in the Sequence Read Archive (SRA) database (accession number: PRJNA1161677). SNP variants were called, and the dataset generated was used to perform a GWAS of HCNp scores in the present study. This dataset has been madeavailable in the Texas Data Repository (https://doi.org/10.18738/T8/RGPPGA).

### 4.6. Data Analysis

#### 4.6.1. Distribution of HCNp Scores and Principal Component Analysis (PCA)

To analyze HCNp scores among different accessions, Tukey’s HSD test was applied using JMP Pro 15 (SAS Institute, Cary, NC, USA). The HCNp scores recorded for both collections were used to generate constellation plots with the software, following the Ward hierarchical method. A PCA was performed using the HCNp scores of the Senegalese accessions from C_1_, along with previously reported seed morphology traits and responses to pathogen inoculations [28,29,30,31]. A partial PCA plot was then constructed to understand these variables’ contribution to the first three principal components (PCs). The relationship between the traits was also investigated through a hierarchical cluster analysis (Ward’s method) using JMP Pro 15. A dendrogram was then constructed.

#### 4.6.2. Correlation Between HCNp and Seed Morphology/Pathogen Inoculation Response Traits

Using the pheatmap package [62] available in the R software (V3.6.0), a clustered Pearson’s correlation heatmap between HCNp levels of the C_1_ accessions and their previously reported traits [28,29,30,31], was created. A heatmap of the distribution of the traits throughout the C_2_ accessions/lines was also generated with pheatmap.

#### 4.6.3. Distribution and Properties of the SNPs in the Sorghum from C_2_

The SNPs generated from the sequences of the C_2_ accessions/lines were analyzed using SNPeff [34] based on the *Sorghum bicolor* reference genome (GCF_000003195.3). The potential locations of these SNPs across the genome and their impacts on genes (e.g., synonymous or nonsynonymous) were predicted.

#### 4.6.4. GWAS

The previously generated SNP dataset [32] was filtered in R (version 4.2.1) to only retain biallelic SNPs using the packages SNPfiltR [63] and vcfR [64]. TASSEL (version 5.2.94) was then used to filter the resulting dataset with the following options: “Site Min Count” = 5, “Site Min Allele Freq” = 0.05, “Site Max Allele Freq” = 0.95, “Min Heterozygous Proportion” = 0.25, and “Max Heterozygous Proportion = 0.75. A filtered dataset of 270,134 SNPs was obtained and used for GWAS. A first analysis was performed using the generalized linear model (GLM) with the average HCNp scores of 112 accessions from C_2_ as phenotypic data, along with the filtered SNPs and their principal components in TASSEL (5.2.94). A subset of the 112 accessions that had their average HCNp scores above 2.55 was used to perform a second GWAS following the same protocol. All the phenotypic data were rank normalized prior to the analyses using the R package RNomni [65]. The output statistics were used to generate Manhattan and Q-Q plots in R (4.2.1) with the R package qqman [66]. Based on the significant SNPs found (*p* = 0.0001), the associated genomic regions were identified using the JBrowse tool available online on Phytozome [67].

#### 4.6.5. RT-qPCR

Welch’s ANOVA test was performed in R (version 4.2.1) using the calculated fold changes. The Games-Howell test was subsequently run using the package rstatix [68]. Compact letter display was obtained with the “cldList” function available in the package rcompanion [69].

## 5. Conclusions

Sorghum is a valuable crop with diverse end uses. The presence of the cyanogenic glucoside dhurrin in this crop has been reported to serve as a barrier against many biotic and abiotic stresses, although high HCN levels are toxic for animal consumption. This study revealed a potential negative relationship between dhurrin, thereby hydrogen cyanide, and SRS infection success on sorghum. This finding will need further validation through testing the expression patterns of the dhurrin catabolic genes upon SRS inoculation. The GWAS conducted revealed significant SNPs located in the same chromosomes harboring the dhurrin biosynthetic and catabolic genes, but not in their vicinity. The use of a larger sample size might increase the SNPs detected, and thus the chances of hits in those regions or nearby. However, most of the significant SNPs detected through GWAS in this study were in unannotated genomic regions, which might be explained by the high number of intergenic SNPs in the dataset used. In addition, GWAS using more samples would help elucidate if HCN release is solely associated with the dhurrin catabolic genes, the biosynthetic genes, or both, thus verifying that these two are proxies of each other as it has long been thought. Assuming further association studies confirm the latter, the findings could serve breeders in the selection of head smut-resistant sorghum in areas where this disease occurs.

## Data Availability

The SNP datasets used in the present study have already been madepublicly available at the Texas Data Repository (TDR) under CC-0 agreement: https://doi.org/10.18738/T8/RGPPGA. The raw sequences of the C_2_ accessions are also available in the Sequence Read Archive (SRA) with the accession number: PRJNA1161677.

## Supplementary Materials

The following supporting information can be downloaded at: https://www.mdpi.com/article/10.3390/plants14050740/s1, Figure S1: QQ-plot from GWAS using the average HCNp scores of 112 accessions/lines from C_2_; Figure S2: QQ-plot from GWAS using the average HCNp scores of C_2_ accessions/lines above 2.55; Figure S3: BLAST results for HS132; Table S1: Information on the accessions used in the study; Table S2: Sequences of the primers and probes used in the RT-qPCR analysis.
