# Supplementary material for: Unveiling the Potential Role of Dhurrin in Sorghum During Infection by the Head Smut Pathogen Sporisorium reilianum f. sp. reilianum
Source: Plants (Basel). 2025 Feb 28;14(5):740. doi: 10.3390/plants14050740 (PMC11901864; doi:10.3390/plants14050740)
Supplement: Supplementary file 1 [file plants-14-00740-s001.zip › plants-3455126-supplementary.pdf]

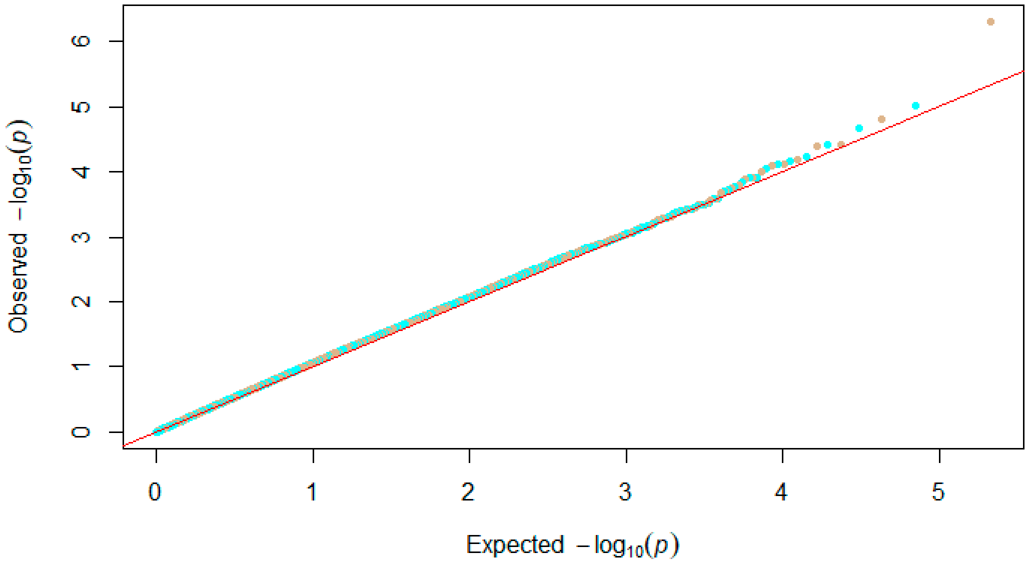

Figure S1: QQ-plot from GWAS using the average HCNp scores of 112 accessions/lines from C_2

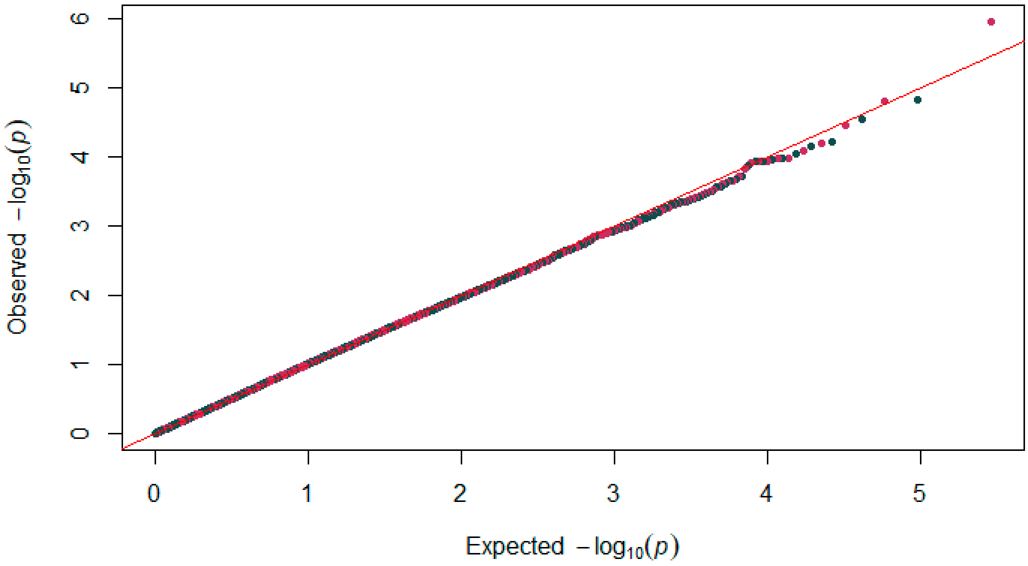

Figure S2: QQ-plot from GWAS using the average HCNp scores of accessions/lines from C_2 above 2.55

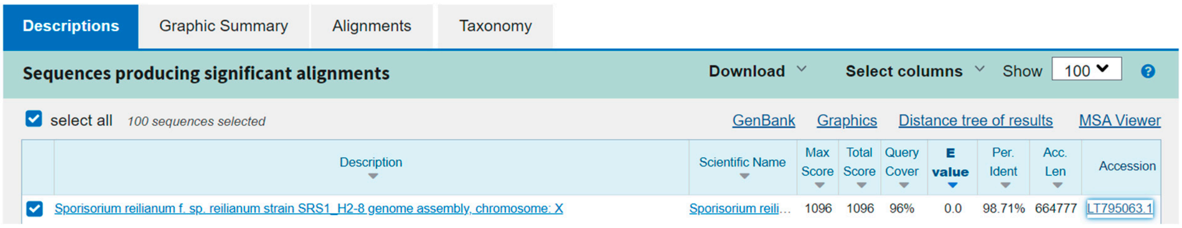

Figure S3: BLAST results using HS132 ITS sequence

Table S1:Detailed information on the accessions used in the study

**ID**	**Name**	**Collection denomination in the paper**	**Race**	**Country of origin**
PI 514279	Fela	C1	Durra	Senegal
PI 514280	Fela	C1	Durra	Senegal
PI 514282	Nieniko	C1	Guinea	Senegal
PI 514283	Fela	C1	Durra	Senegal
PI 514284	Nieniko	C1	Guinea	Senegal
PI 514285	Fela	C1	Durra	Senegal
PI 514286	Fela	C1	Durra	Senegal
PI 514287	Fela	C1	Durra	Senegal
PI 514288	Fela	C1	Durra	Senegal
PI 514289	Nieniko	C1	Guinea	Senegal
PI 514290	Bassi	C1	Guinea	Senegal
PI 514291	Nieniko	C1	Guinea	Senegal
PI 514292	Saban	C1	Durra	Senegal
PI 514293	Nieniko	C1	Guinea	Senegal
PI 514294	Nieniko	C1	Guinea	Senegal
PI 514295	Nieniko	C1	Guinea	Senegal

PI 514296	Nieniko	C1	Guinea	Senegal
PI 514297	Nieniko	C1	Guinea	Senegal
PI 514298	Nieniko	C1	Guinea	Senegal
PI 514299	Nieniko	C1	Guinea	Senegal
PI 514300	Nieniko	C1	Guinea	Senegal
PI 514301	Bassi	C1	Guinea	Senegal
PI 514302	Nieniko	C1	Guinea	Senegal
PI 514303	Nieniko	C1	Guinea	Senegal
PI 514304	Nieniko	C1	Guinea	Senegal
PI 514305	Nieniko	C1	Guinea	Senegal
PI 514306	Nieniko	C1	Guinea	Senegal
PI 514307	Nieniko	C1	Guinea	Senegal
PI 514308	Nieniko	C1	Guinea	Senegal
PI 514309	Nieniko	C1	Guinea	Senegal
PI 514310	Nieniko	C1	Guinea	Senegal
PI 514311	Nieniko	C1	Guinea	Senegal
PI 514312	Nieniko	C1	Guinea	Senegal
PI 514313	Nieniko	C1	Guinea	Senegal
PI 514314	Nieniko	C1	Guinea	Senegal
PI 514316	Nieniko Sambo Marr	C1	Guinea	Senegal
PI 514317	Nieniko Harri	C1	Guinea	Senegal
PI 514318	Nieniko	C1	Guinea	Senegal
PI 514319	Nieniko	C1	Guinea	Senegal

PI 514320	Nieniko	C1	Guinea	Senegal
PI 514321	Nieniko	C1	Guinea	Senegal
PI 514322	Nieniko	C1	Guinea	Senegal
PI 514323	Same	C1	Durra	Senegal
PI 514324	Fela	C1	Durra	Senegal
PI 514325	Nieniko	C1	Guinea	Senegal
PI 514326	Nieniko	C1	Guinea	Senegal
PI 514332	Bassi	C1	Guinea	Senegal
PI 514333	Bassi	C1	Guinea	Senegal
PI 514334	Bassi Bambara	C1	Guinea	Senegal
PI 514335	Bassi	C1	Guinea	Senegal
PI 514336	Bassi	C1	Guinea	Senegal
PI 514337	Bassi Tourka	C1	Guinea	Senegal
PI 514338	Bassi Tourka	C1	Guinea	Senegal
PI 514339	Bassi Yathiar	C1	Guinea	Senegal
PI 514340	Bassi Tourka	C1	Guinea	Senegal
PI 514341	Bassi Yathiar	C1	Guinea	Senegal
PI 514342	Bassi Tin	C1	Guinea	Senegal
PI 514343	Bassi Kinte	C1	Guinea	Senegal
PI 514344	Bassi Gaoya	C1	Guinea	Senegal
PI 514345	Bassi Kaba	C1	Guinea	Senegal
PI 514346	Bassi	C1	Guinea	Senegal
PI 514347	Bassi Wende	C1	Guinea	Senegal

PI 514348	Bassi Wende	C1	Guinea	Senegal
PI 514349	Bassi Wende	C1	Guinea	Senegal
PI 514350	Bassi Wende	C1	Guinea	Senegal
PI 514351	Bassi Wende	C1	Guinea	Senegal
PI 514352	Congossane	C1	Guinea	Senegal
PI 514353	Congossane	C1	Guinea	Senegal
PI 514354	Bassi Tchangal	C1	Guinea	Senegal
PI 514355	Bassi Tin	C1	Guinea	Senegal
PI 514356	Bassi Kinte	C1	Guinea	Senegal
PI 514360	Bassi	C1	Guinea	Senegal
PI 514361	Bassi	C1	Guinea	Senegal
PI 514362	Bassi	C1	Guinea	Senegal
PI 514363	Bassi	C1	Guinea	Senegal
PI 514364	Bassi	C1	Guinea	Senegal
PI 514366	Congossane	C1	Guinea	Senegal
PI 514367	Fela	C1	Durra	Senegal
PI 514368	Bassi	C1	Guinea	Senegal
PI 514371	Bassi	C1	Guinea	Senegal
PI 514372	Nieniko	C1	Guinea	Senegal
PI 514373	Bassi	C1	Guinea	Senegal
PI 514374	Congossane	C1	Guinea	Senegal
PI 514375	Bassi	C1	Guinea	Senegal
PI 514376	Bayeri	C1	Guinea	Senegal

PI 514377	Bassi Tchangi	C1	Guinea	Senegal
PI 514378	Bassi Tourka	C1	Guinea	Senegal
PI 514379	Bassi Nebamaro	C1	Guinea	Senegal
PI 514380	Nieniko	C1	Guinea	Senegal
PI 514381	Congossane	C1	Guinea	Senegal
PI 514382	Nieniko	C1	Guinea	Senegal
PI 514383	Sambatako	C1	Guinea	Senegal
PI 514387	Bassi Nebamaro	C1	Guinea	Senegal
PI 514388	Nieniko	C1	Guinea	Senegal
PI 514390	Nieniko	C1	Guinea	Senegal
PI 514391	Nieniko	C1	Guinea	Senegal
PI 514392	Nieniko Sobak	C1	Guinea	Senegal
PI 514393	Amadi Bamba	C1	Guinea	Senegal
PI 514394	Bassi	C1	Guinea	Senegal
PI 514395	Bassi	C1	Guinea	Senegal
PI 514396	Nieniko	C1	Guinea	Senegal
PI 514397	Bayeri	C1	Guinea	Senegal
PI 514398	Bayeri	C1	Guinea	Senegal
PI 514399	Nieniko	C1	Guinea	Senegal
PI 514400	Nieniko Harri	C1	Guinea	Senegal
PI 514401	Nieniko Harri	C1	Guinea	Senegal
PI 514403	Nieniko	C1	Guinea	Senegal
PI 514404	Nagbani	C1	Durra	Senegal

PI 514405	Guadiaba	C1	Durra	Senegal
PI 514409	Bassi	C1	Guinea	Senegal
PI 514411	Nieniko	C1	Guinea	Senegal
PI 514412	Nieniko Yabiti	C1	Guinea	Senegal
PI 514414	Nienikel	C1	Guinea	Senegal
PI 514417	Nienikel	C1	Guinea	Senegal
PI 514418	Nieniko	C1	Guinea	Senegal
PI 514419	Gadyabo	C1	Guinea	Senegal
PI 514420	Dambayele	C1	Guinea	Senegal
PI 514423	Nienikel	C1	Guinea	Senegal
PI 514424	Bayeri	C1	Guinea	Senegal
PI 514425	Sambadyabo	C1	Guinea	Senegal
PI 5144126	Sambadyabo	C1	Guinea	Senegal
PI 514427	Bayeri	C1	Guinea	Senegal
PI 514428	Nieniko	C1	Guinea	Senegal
PI 514429	Nieniko	C1	Guinea	Senegal
PI 514430	Bayeri	C1	Guinea	Senegal
PI 514431	Nieniko	C1	Guinea	Senegal
PI 514432	Nienikel	C1	Guinea	Senegal
PI 514433	Amadi Bamba	C1	Guinea	Senegal
PI 514434	Bayeri	C1	Guinea	Senegal
PI 514435	Nieniko	C1	Guinea	Senegal
PI 514436	Nienikel	C1	Guinea	Senegal

PI 514437	Bassi	C1	Guinea	Senegal
PI 514438	Nienikel	C1	Guinea	Senegal
PI 514439	Nienikel	C1	Guinea	Senegal
PI 514440	Amadi Bamba	C1	Guinea	Senegal
PI 514444	Bodya	C1	Guinea	Senegal
PI 514446	Nienikel	C1	Guinea	Senegal
PI 514448	Nieniko	C1	Guinea	Senegal
PI 514449	Kinto	C1	Guinea	Senegal
PI 514452	Nieniko	C1	Guinea	Senegal
PI 514453	Bayeri	C1	Guinea	Senegal
PI 514454	Amadi Bamba	C1	Guinea	Senegal
PI 514455	Makabe	C1	Caudatum	Senegal
PI 514456	N/A	C1	Guinea	Senegal
PI 514457	Kinto	C1	Guinea	Senegal
PI 514458	Gadyabo	C1	Caudatum	Senegal
PI 514459	Makanio Sune	C1	Bicolor	Senegal
PI 514460	Kinto	C1	Guinea	Senegal
PI 514461	Makabe	C1	Caudatum	Senegal
PI 514462	Kinto	C1	Guinea	Senegal
PI 514463	Makabe	C1	Caudatum	Senegal
PI 514464	Gadyabo	C1	Caudatum	Senegal
PI 514465	Kinto	C1	Guinea	Senegal
PI 514466	Kinto	C1	Guinea	Senegal

PI 514467	Nio	C1	Guinea	Senegal
PI 514468	Kinto	C1	Guinea	Senegal
PI 514469	Nio	C1	Guinea	Senegal
PI 514471	Kinto	C1	Guinea	Senegal
PI 514472	Nio	C1	Guinea	Senegal
PI 514473	Kinto	C1	Guinea	Senegal
PI 514474	Nio	C1	Guinea	Senegal
PI 514475	Nio	C1	Guinea	Senegal
PI 514478	Nio	C1	Guinea	Senegal
S1	Tigne	C2	Not available	Senegal
S2	Goor Gatte	C2	Not available	Senegal
S3	Tegne	C2	Not available	Senegal
S4	Darou	C2	Not available	Senegal
S5	E48	C2	Not available	Senegal
S6	Fehla E2	C2	Not available	Senegal
S7	Makebe	C2	Not available	Senegal
S8	Bou Gatte	C2	Not available	Senegal
S9	Mbodiene	C2	Not available	Senegal
S10	Sorgho	C2	Not available	Senegal
S11	Tegne #1	C2	Not available	Senegal
S12	Nioo	C2	Not available	Senegal
S13	Tegne #2	C2	Not available	Senegal
S14	Negne	C2	Not available	Senegal

S15	E05	C2	Not available	Senegal
S16	E32	C2	Not available	Senegal
S17	E63	C2	Not available	Senegal
S18	E54	C2	Not available	Senegal
S19	E38	C2	Not available	Senegal
S20	E40	C2	Not available	Senegal
S21	E60	C2	Not available	Senegal
S22	E64	C2	Not available	Senegal
S23	E07	C2	Not available	Senegal
S24	E02	C2	Not available	Senegal
S25	E04	C2	Not available	Senegal
S26	Tendji	C2	Not available	Senegal
S27	E47	C2	Not available	Senegal
S28	E41	C2	Not available	Senegal
S29	E23	C2	Not available	Senegal
S30	Golobe	C2	Not available	Senegal
S31	Naga White	C2	Not available	Senegal
S32	Goor Gatte E93	C2	Not available	Senegal
S33	Nganda	C2	Not available	Senegal
S34	Nguinthe	C2	Not available	Senegal
S35	Mbodiene E89	C2	Not available	Senegal
S36	Ngatte	C2	Not available	Senegal
S37	CE 180-33	C2	Not available	Senegal

S38	Sureno	C2	Not available	Senegal
S39	Ouregne	C2	Not available	Senegal
S40	Nioo E125	C2	Not available	Senegal
S41	Pathie	C2	Not available	Senegal
S42	F2-20	C2	Not available	Senegal
S43	Bougatte E71	C2	Not available	Senegal
S44	Tigne E102	C2	Not available	Senegal
S45	SK 5912 short Koura	C2	Not available	Senegal
S46	CE 151-262	C2	Not available	Senegal
S47	Bassi	C2	Not available	Senegal
S48	CE 145-66	C2	Not available	Senegal
S49	Sorvato 1	C2	Not available	Senegal
S50	Faourou	C2	Not available	Senegal
S51	E39	C2	Not available	Senegal
S52	Macia	C2	Not available	Senegal
S53	Grinkan	C2	Not available	Senegal
S54	Bassi Bou Wekh	C2	Not available	Senegal
S55	Baasi Bou Wekh E6	C2	Not available	Senegal
S56	Pim Bou Wekh	C2	Not available	Senegal
S57	Kinty	C2	Not available	Senegal
S58	Payenne	C2	Not available	Senegal
S59	tenya	C2	Not available	Senegal
S60	CE 196-7-2-1	C2	Not available	Senegal

N1	Maradi	C2	Not available	Niger
N2	Maradi Dakwara	C2	Not available	Niger
N3	Maradi Voyage	C2	Not available	Niger
N4	Maradi Bakin Birgi	C2	Not available	Niger
N5	Maradi Dan Gao	C2	Not available	Niger
N6	Maradi Karin Kapani	C2	Not available	Niger
N7	Maradi Koura Mota	C2	Not available	Niger
N8	Maradi Kallarwaran	C2	Not available	Niger
N9	Maradi Guidan Bouzaye	C2	Not available	Niger
N10	Maradi kadata	C2	Not available	Niger
N11	Maradi Birnin Lalle	C2	Not available	Niger
N12	Maradi Mountarou Barmo	C2	Not available	Niger
N13	Maradi Sarkin Diya	C2	Not available	Niger
N14	Maradi Dan Toumbi	C2	Not available	Niger
N15	Mota Maradi	C2	Not available	Niger
N16	Maradi Guidan Karo	C2	Not available	Niger
N17	Maradi Garin Mahaman	C2	Not available	Niger
N18	Tagabati Mota Maradi	C2	Not available	Niger
N19	Maradi Jaja	C2	Not available	Niger
N20	Maradi Karo Saboua	C2	Not available	Niger
N21	Tahoua Guidan Daouda	C2	Not available	Niger
N22	Tahoua Tamske	C2	Not available	Niger
N23	Tahoua Guidan Faji	C2	Not available	Niger

N24	Tahoua Boulaya	C2	Not available	Niger
N25	Tahoua Moujia	C2	Not available	Niger
N26	Tahoua Toumbou Gana	C2	Not available	Niger
N27	Tahoua Guidan Boutou	C2	Not available	Niger
N28	Tahoua Bazaga	C2	Not available	Niger
N29	Tahoua Magaria Tacha	C2	Not available	Niger
N30	Tahoua Tacha Illias	C2	Not available	Niger
N31	Tahoua Madaoua	C2	Not available	Niger
N32	Tahoua Gorangg	C2	Not available	Niger
N33	Koirategui Bangue	C2	Not available	Niger
N34	Tagabati BDF	C2	Not available	Niger
N35	Laboda	C2	Not available	Niger
N36	Gauwa	C2	Not available	Niger
N37	Dosso Bouvami	C2	Not available	Niger
N38	Sina Koira	C2	Not available	Niger
N39	Cochila Bella #1	C2	Not available	Niger
N40	Kochilan Bella	C2	Not available	Niger
N41	Tillaberi	C2	Not available	Niger
N42	Till IRAT 204	C2	Not available	Niger
N43	Station de Tarna 5-35	C2	Not available	Niger
N44	Tagabati Liddi	C2	Not available	Niger
N45	Hamokoiray: Karrin Haoussa	C2	Not available	Niger
N46	Haoussa	C2	Not available	Niger

N47	Kangnamalam (Zinder)	C2	Not available	Niger
N48	Tillaberi #1	C2	Not available	Niger
N49	Tahadoua	C2	Not available	Niger
N50	Gogazey	C2	Not available	Niger
N51	Kochitan Bella #1	C2	Not available	Niger
N52	Madaoua	C2	Not available	Niger
N53	Tchoudaoria	C2	Not available	Niger
N54	Koira Tegui Bangou	C2	Not available	Niger
N55	HKH	C2	Not available	Niger
N56	Damana	C2	Not available	Niger
N57	Koni	C2	Not available	Niger
N58	Maourey	C2	Not available	Niger
N59	Koria Hassa	C2	Not available	Niger
N60	Sokomba Kodo	C2	Not available	Niger

Table S2: Sequences of primers and probes used in the study

Gene	Oligo type	Forward sequence	Reverse Sequence	Length	Tm	GC %
Actin-1	Primer	GCATTCACGAGACTACCTACAA	NA	22	62	45.5
Actin-1	Primer	NA	CAGCAATCCCAGGGAACATA	20	62	50
Actin-1	Probe	ATGGCAACATCGTCCTCTCTGGTG	NA	24	68	54
CYP79A1	Primer	CCAAGAGCGACACCTTCAT	NA	19	62	52.6
CYP79A1	Primer	NA	GACCGGCCGTACGTTTAAT	19	62	52.6
CYP79A1	Probe	ACCTCTACCCGTCCATCTCCATCT	NA	24	68	54
CYP71E1	Primer	GACTGAATAAGCGTGCCAAAG	NA	21	62	47.6
CYP71E1	Primer	NA	GATAGCTCCAACAGTGTCCAA	21	62	47.6
CYP71E1	Probe	ACGTACAGCTAGCCAACAGTCAGA	NA	24	67	50
UGT85B1	Primer	CCTCATCCTCAACACCCTGTA	NA	21	63	52.4
UGT85B1	Primer	NA	TGTAGATCGGCGGGAAGAA	19	63	52.6

UGT85B1	Probe	AGCTCGAGAAGGACGTGGTGGA	NA	22	69	59
